# ZCURVE 3.0: identify prokaryotic genes with higher accuracy as well as automatically and accurately select essential genes

**DOI:** 10.1093/nar/gkv491

**Published:** 2015-05-14

**Authors:** Zhi-Gang Hua, Yan Lin, Ya-Zhou Yuan, De-Chang Yang, Wen Wei, Feng-Biao Guo

**Affiliations:** 1 Center of Bioinformatics, School of Life Science and Technology, University of Electronic Science and Technology of China, Chengdu 610054, China; 2 Center for Information in BioMedicine, University of Electronic Science and Technology of China, Chengdu 610054, China; 3 Health Big Data Science Research Center, Big Data Research Center, University of Electronic Science and Technology of China, Chengdu 610054, China; 4 Key Laboratory for NeuroInformation of the Ministry of Education, University of Electronic Science and Technology of China, Chengdu 610054, China; 5 Department of Physics, Tianjin University, Tianjin 300072, China; 6 Key Laboratory of Systems Bioengineering, Ministry of Education, Tianjin 300072, China; 7 Collaborative Innovation Center of Chemical Science and Engineering, Tianjin 300072, China

## Abstract

In 2003, we developed an *ab initio* program, ZCURVE 1.0, to find genes in bacterial and archaeal genomes. In this work, we present the updated version (i.e. ZCURVE 3.0). Using 422 prokaryotic genomes, the average accuracy was 93.7% with the updated version, compared with 88.7% with the original version. Such results also demonstrate that ZCURVE 3.0 is comparable with Glimmer 3.02 and may provide complementary predictions to it. In fact, the joint application of the two programs generated better results by correctly finding more annotated genes while also containing fewer false-positive predictions. As the exclusive function, ZCURVE 3.0 contains one post-processing program that can identify essential genes with high accuracy (generally >90%). We hope ZCURVE 3.0 will receive wide use with the web-based running mode. The updated ZCURVE can be freely accessed from http://cefg.uestc.edu.cn/zcurve/ or http://tubic.tju.edu.cn/zcurveb/ without any restrictions.

## INTRODUCTION

Proteins perform many functions in organisms, and they are fundamental for every cell. Protein-coding genes are recognized in sequenced genomes by three methods (1). First, the experimental transcription track may be the most specific method with the fewest false-positive results. However, this method is only applicable to constitutively expressed (housekeeping) genes and those expressed at the investigated experimental stage. In comparison, genes that are expressed in limited conditions will be missed by this method. Similarly, a search using basic local alignment search tool (BLAST) is another effective method to recognize genes. Homologs of known genes in public databases could be selected using this method. However, strain-specific genes would be missed, and there may be a large problem when sequenced strains do not have any close relatives in public databases. Finally, composition-based *ab initio* constitutes the most commonly used method (2). With more and more genomes being sequenced, this method increasingly plays an important role in the field of gene annotation.

In 2003, we published an *ab initio* gene-calling program named ZCURVE 1.0 (3), which can be used independently or jointly with other programs to annotate bacterial and archaeal genomes; this program is based on the Z-curve theory of DNA sequence (4). So far, ZCURVE 1.0 has over 100 registered users and has been used in dozens of genome-sequencing projects of prokaryotes (5). The original publication describing ZCURVE 1.0 has attracted 170 citations in the past 12 years according to Google scholar. For example, in their very excellent work, Egan and colleagues used ZCURVE 1.0 to predict coding potentials to support their functional analysis (6). In this work, we updated the ZCURVE program to version 3.0 through extending its function by appending a new module and improving the accuracy by modifying the algorithm.

## MATERIALS AND METHODS

### ZCURVE algorithm improvement

Compared with ZCURVE 1.0, ZCURVE 3.0 has been improved in the following four aspects. First, in the original version, we only considered the frequencies of codon-position-dependent single nucleotides and the frequencies of phase-specific dinucleotides occurring at the codon positions 1–2 and 2–3 and transformed them into the *Z* format (4 × 3 × 3/4 + 4^2^ × 2 × 3/4 = 9 + 24 = 33 variables). To extract more information and achieve higher accuracy, currently we further consider the short-term correlation among three or four adjacent nucleotides. That is to say, frequencies of 192 (4^3^ × 3) phase-specific trinucleotides are transformed into 144 (192 × 3/4) *Z* variables and frequencies of 768 (4^4^ × 3) phase-specific tetranucleotides are transformed into 576 (768 × 3/4) *Z* variables. In addition, the frequencies of phase-specific dinucleotides occurring at the codon positions 3–1 are also taken into account and there will be 36 (4^2^ × 3 × 3/4) corresponding variables for dinucleotides. Hence, the number of characteristic variables changed from 33 to 765 (9 + 36 + 144 + 576) in ZCURVE 3.0. The *Z* transformation is responsible to change the four nucleotides into three distributions of purine versus pyrimidine, amino versus keto, and weak hydrogen bonds versus strong hydrogen bonds. If you want to learn more details and see the mathematical formula about the transformation, please refer to (7).

Second, the Fisher linear discriminant was used as the classifier to differentiate genes and non-coding ORFs. In ZCURVE 3.0, instead the most widely used machine learning algorithm, support vector machine is employed, which has shown excellent performance in many classifying problems, particularly when both the positive and negative sample groups have a balanced size. In our program, the SVM-light toolbox is used and it could be freely available at http://svmlight.joachims.org/ (8). Among the four provided kernel functions, linear kernel is chosen because of the larger number of variables. For all the other parameters, their default settings are used.

Third, as a newly added function, ZCURVE 3.0 has the capacity to select the most important subset-essential genes from the complete list of genes in a certain genome. Essential genes are critical for the survival of an organism under any condition, and they play a significant role in pharmaceutics and synthetic biology (9). Usually, essential genes are experimentally determined in optimal growth condition. Because of the high cost and huge amount of labor required to determine essential genes by wet experiments, researchers recognized that computational prediction may be an easy and feasible method (10). Furthermore, experimental results may bias to some extent because there may be controversy among groups in deciding what are the most favorable growing conditions (10). Our research group developed an automatic program to predict essential genes in prokaryotic genomes (11). Geptop combines results of comparative genomics from diverse reference sets by weighting them with evolutionary distances (11). Through the weighting integration, it achieves significantly higher accuracy than previous algorithms for predicting prokaryotic essential genes (12). Tests using 19 prokaryotes show that the area under operation character curve (AUC) ranged from 0.569 to 0.978 (please refer to Figure 5 of (11) for the details). The AUC is as high as 97.8% and 95.2% in the two most thoroughly investigated model genomes, *Escherichia coli* MG1655 and *Bacillus subtilis* 168, which have the most accurate genome-scale experimental essentiality data. The Geptop program (11) is embedded within the ZCURVE 3.0 package as a post-processing tool. Currently, ZCURVE is the only available program that can automatically select essential genes from predicted gene list.

Fourth, the old version of our program is a standalone tool that needed to be downloaded from the Tubic website. To facilitate its use, we have provided a web-based running mode for the latest version. Users can freely and easily use the program by visiting either of our two websites, Tubic or Cefg.

Like the old version, ZCURVE 3.0 uses the subprogram GS-finder to assign translation start sites for predicted genes. It has rather reliable predictions of translation start sites and correctly assigned 90% of 5′ termini in experimental sets from *E. coli* and *B. subtilis* (13). A recent review (14) indicated that GS-finder could generate better results than RBS-finder, which is used by Glimmer. Alternatively, the users could deal with the 5′ terminus of the ZCURVE results using some other post-processing tools such as TriTISA (15).

### Input and output of ZCURVE 3.0 web service

**Figure 1.**
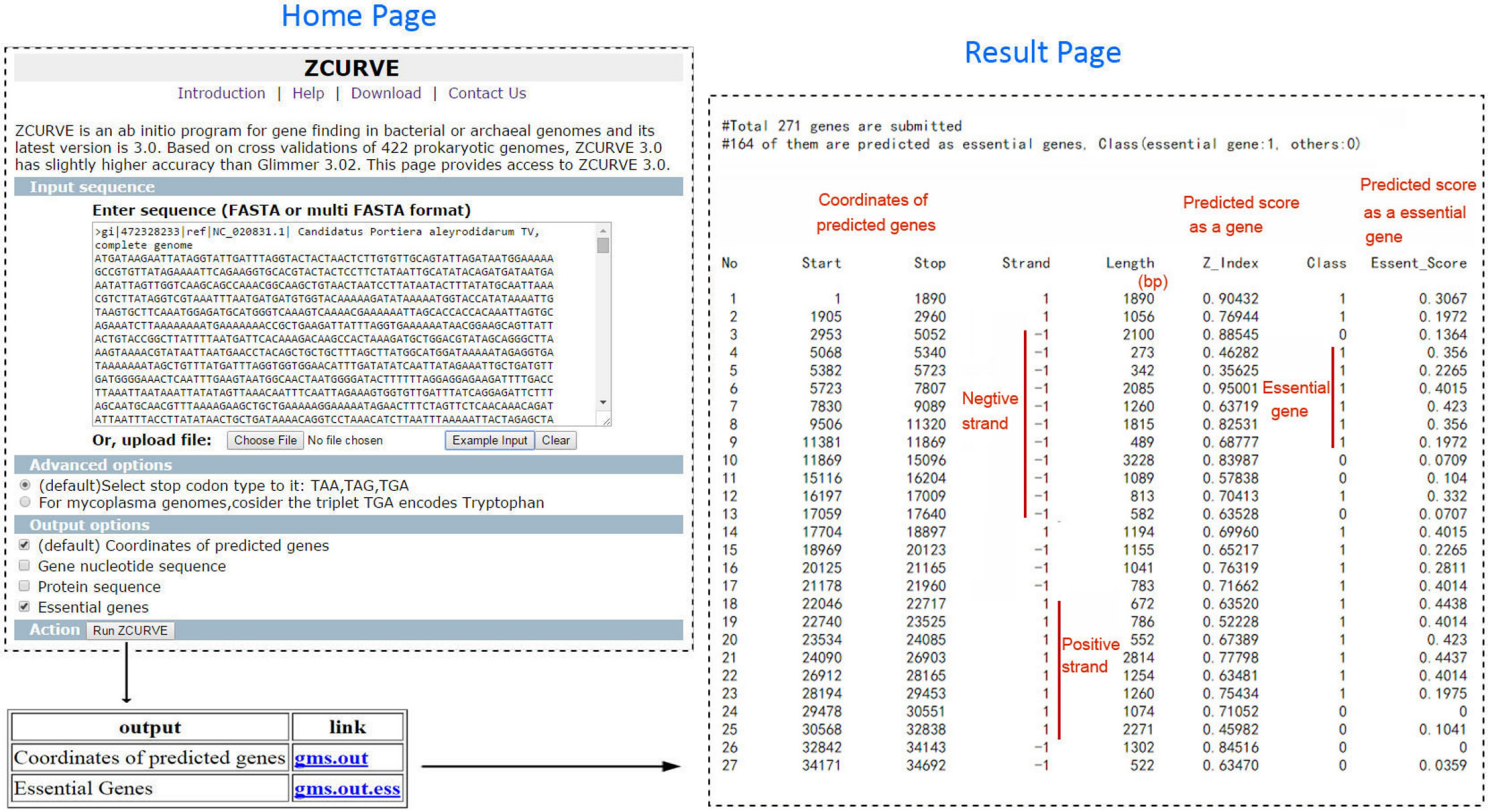
Submission interface (left top panel), option menu (left bottom panel), and an example of output information (right panel) using the *Portiera aleyrodidarum* genome.

For the input, we provide an example of the *Portiera aleyrodidarum* TV (NC_020831) genome on our ZCURVE web server, which has a very small genome size that enables a quick loading time. When annotating one anonymous genome, the users must prepare a standard FASTA sequence file as well as the example. Also, our server can handle multiple FASTA-formatted sequences, which is especially useful when users submit contigs of a draft genome. Our program can output four files by selecting each of the four output options. One is the chromosomal coordinates of all predicted genes. The second file includes DNA sequences of all genes in FASTA format. The third file corresponds to amino acid sequences encoded by each predicted gene. The last output file contains the essentiality of all predicted genes provided by our post-processing program Geptop. The essentiality score indicates the importance of the gene function: the higher the essentiality score, the more important the gene function. The first three files are output immediately (usually within several seconds), whereas the essentiality information needs over one hour to output. Therefore, users must record the outputting link and view the results two or three hours later. Figure 1 illustrates the submission interface, option menu, and one example of the output for the coding potential and essentiality prediction.

### Indexes to evaluate the performance of ZCURVE 3.0

The performance of ZCURVE 3.0 in the prediction of coding potentials and essentiality was evaluated in this work. To measure coding potentials prediction, we used the following indexes:

(1) \begin{equation*} \begin{array}{*{20}l} {{\rm Sensitivity}\,(S_n ) = } \\ {\frac{\displaystyle{{\rm the}\;{\rm number}\;{\rm of}\;{\rm correctly}\;{\rm predicted}\;{\rm genes}\;{\rm in}\;{\rm one}\;{\rm genome}}}{\displaystyle{{\rm the}\;{\rm number}\;{\rm of}\;{\rm all}\;{\rm annotated}\;{\rm genes}\;{\rm in}\;{\rm the}\;{\rm genome}}}} \\ \end{array} \end{equation*}

(2) \begin{equation*} \begin{array}{*{20}l} {{\rm Pecision}\,({\rm PPV}) = } \\ {\frac{\displaystyle{{\rm the}\;{\rm number}\;{\rm of}\;{\rm correctly}\;{\rm predicted}\;{\rm genes}\;{\rm in}\;{\rm the}\;{\rm genome}}}{\displaystyle{{\rm the}\;{\rm number}\;{\rm of}\;{\rm all}\;{\rm predicted}\;{\rm genes}\;{\rm for}\;{\rm the}\;{\rm genome}}}} \\ \end{array} \end{equation*}

Because negative samples could not be determined in a test set that was used to evaluate coding potential, the precision index was adopted instead of the specificity index. Hence, we define the accuracy index as follows:

(3) \begin{equation*} {\rm Accuracy} = \frac{{S_n + {\rm PPV}}}{2} \end{equation*}

We also defined the following index to denote the percentage of additional prediction:

(4) \begin{equation*} \begin{array}{*{20}l} {{\rm Additional}\;{\rm positive}\;{\rm rate}\,({\rm APR}) = } \\ {\frac{\displaystyle{{\rm the}\;{\rm number}\;{\rm of}\;{\rm additionally}\;{\rm predicted}\;{\rm genes}}}{\displaystyle{{\rm the}\;{\rm number}\;{\rm of}\;{\rm annotated}\;{\rm genes}}}} \\ \end{array} \end{equation*}

Note that the prediction is better when the former three indexes are higher. However, a low value is better for additional positive rate.

The numbers of essential genes and non-essential genes are extremely imbalanced. For example, there are only 288 essential genes in the *E. coli* MG1655 genome; however, the number of non-essential genes is as large as 3852. For this special case, we primarily used the integrated index to evaluate the essentiality prediction of our program.

(5) \selectfont\begin{equation*} \begin{array}{*{20}l} {{\rm Overall}\;{\rm accuracy}\,({\rm OA}) = } \\ {\frac{\displaystyle{{\rm the}\;{\rm number}\;{\rm of}\;{\rm correctly}\;{\rm predicted}\;{\rm essential}\;{\rm genes}\;plus\;{\rm nonessential}\;{\rm genes}}}{\displaystyle{{\rm the}\;{\rm total}\;{\rm number}\;{\rm of}\;{\rm both}\;{\rm essential}\;{\rm genes}\;{\rm and}\;{\rm nonessential}\;{\rm genes}}}} \\ \end{array} \end{equation*}

The commonly used indexes, sensitivity and specificity, are also used as additional references.

(6) \selectfont\begin{equation*} \begin{array}{*{20}l} {S_n = } \\ {\frac{\displaystyle{{\rm the}\;{\rm number}\;{\rm of}\;{\rm correctly}\;{\rm predicted}\;{\rm essential}\;{\rm genes}}}{\displaystyle{{\rm the}\;{\rm number}\;{\rm of}\;{\rm all}\;{\rm annotated}\;{\rm essential}\;{\rm genes}\;{\rm for}\;{\rm the}\;{\rm genome}}}} \\ \end{array} \end{equation*}

(7) \selectfont\begin{equation*} S_p = \frac{\displaystyle{{\rm the}\;{\rm number}\;{\rm of}\;{\rm correctively}\;{\rm predicted}\;{\rm nonessential}\;{\rm genes}}}{\displaystyle{{\rm the}\;{\rm number}\;{\rm of}\;{\rm all}\;{\rm annotated}\;{\rm nonessential}\;{\rm genes}\;{\rm for}\;{\rm the}\;{\rm genome}}} \end{equation*}

A gene would only be considered a correct prediction if ZCURVE 3.0 not only found the gene but also correctly decided its essentiality. Experimentally determined essential genes in optimal growing conditions were taken as the benchmark of testing our program.

## RESULTS AND DISCUSSION

### The performance of ZCURVE 3.0 on 422 completely sequenced genomes

To evaluate the performance of ZCURVE 3.0 in the prediction of coding potentials of prokaryotic genes, we selected 422 genomes that were sequenced before 2007 that did not use the ZCURVE program in the annotating process so that the genomes could be used as a strict test set. For these genomes, we downloaded ‘*.fna’ and ‘*.ptt’ files from the RefSeq site at the National Center for Biotechnology Information (NCBI) (16). A gene could be considered a correct prediction only if the prediction and annotated gene had a consistent 3′ terminus. Results for each genome are listed in Supplementary Table S1 and are summarized in Table 1. As demonstrated, the sensitivity and precision of ZCURVE 3.0 were 95.3% and 92.1%, respectively, whereas those of ZCURVE 1.0 were 95.5% and 82.0%. This indicates that our updated version has an accuracy (93.7%) that was 5% higher than that of the original version (88.7%). Conversely, Glimmer 3.02 (17), which is the most common *ab initio* prokaryotic gene finder, has an accuracy of 93.0%. In detail, Glimmer 3.0 has 1.0% higher sensitivity than ZCURVE 3.0. However, the latter has 2.5% higher precision than the former. Therefore, we can safely conclude that our program gives similar performance with Glimmer in the identification of protein-coding genes. Note that the standard deviation of the accuracies for Glimmer 3.02 is 1.3% higher than that of ZCURVE 3.0, indicating that ZCURVE may have more robust results than Glimmer. In fact, Glimmer produced poor results for a few genomes, whereas ZCURVE did not. For example, the sensitivity of Glimmer 3.02 was as low as 56.9% for the genome *Anaeromyxobacter dehalogenans* (NC_007760), and the precision was only 62.4%. In contrast, ZCURVE 3.0 gave stable results for this genome, with a sensitivity of 94.3% and precision of 95.7%. As another example, the precision of Glimmer 3.02 was only 44.8% for the *Trichodesmium erythraeum* (NC_008312) genome, whereas ZCURVE yielded a precision of 87.2%; both programs yielded a sensitivity of ∼94%.

**Table 1.** General prediction results of three programs with 422 prokaryotic genomes

	*S_n_*: mean (SD)	*PPV*: mean (SD)	Accuracy: mean (SD)
ZCURVE 3.0	0.9525 (0.03253)	0.9212 (0.06026)	0.9368 (0.03563)
ZCURVE 1.0	0.9549 (0.05742)	0.8195 (0.1154)	0.8872 (0.07218)
Glimmer 3.02	0.9625 (0.03452)	0.8965 (0.08235)	0.9295 (0.04783)

*S_n_*, sensitivity; *PPV*, precision; SD, standard deviation.

We also used ZCURVE 3.0 to analyze 2787 bacterial and archaeal genomes that were sequenced between the year 2007 and 2013. Consequently, the average sensitivity of ZCURVE 3.0 was 95.1%, and the precision was 92.6% (Supplementary Table S2). The accuracy, which is the mean value of the above two indexes, should be 93.9%. Therefore, ZCURVE 3.0 gives stable results using large test sets. Finally, the quality of the annotation for each of the 422 genomes differs because of the applied annotation strategy. Therefore, the factual accuracy of both Glimmer and ZCURVE might be higher than that reported here because a few errors (particularly hypothetical genes) may exist in the RefSeq annotation files for some genomes, which have been used as the gold standard by us.

### Joint application of ZCURVE 3.0 and Glimmer 3.02

**Figure 2.**
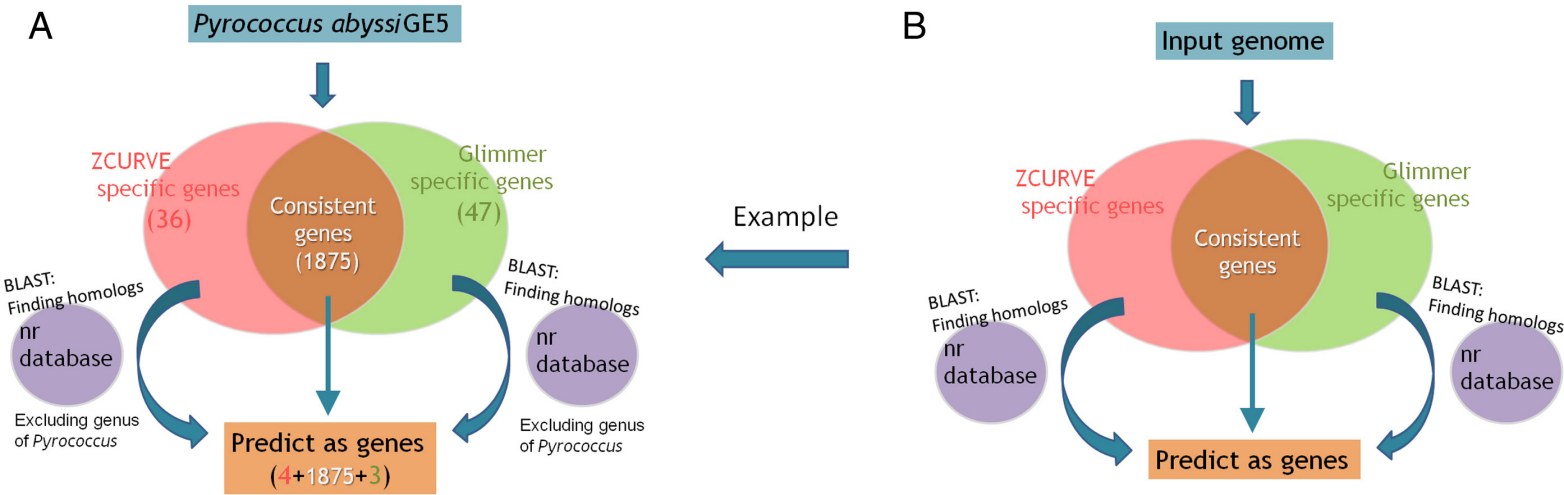
Sketch of the procedure for the joint application of ZCURVE 3.0 and Glimmer 3.02. (**A**) A simulated example using the *Pyrococcus abyssi* GE5 genome. (**B**) Suggested pipeline when using a newly sequenced genome.

We do not intend to replace the Glimmer system with our ZCURVE program. Instead, we wish to provide an alternative solution for the issue of prokaryotic gene finding and hope it could be complementary to Glimmer. In fact, ZCURVE and Glimmer are based on different principles. Glimmer is a Markov-chain–based method, which reflects local statistical characteristics of coding sequences, whereas ZCURVE is based mainly on global statistical characteristics of coding sequences (3). Therefore, the two algorithms are thought to be complementary. However, they would provide basically consistent predictions for conserved genes because they share the same aim. There may be a few differences between the predictions of species-specific genes. Note that these genes compose only a small fraction of the gene collective. Combining the two gene finders may generate improved prediction to some extent. Here, we randomly chose 50 genomes for which ZCURVE 3.0 and Glimmer 3.02 predictions yielded similar accuracy to illustrate the results of joint predictions. The combining strategy is detailed as follows. First, each program generated its output. There were many consistent predictions between the results of the two programs. These overlaps were directly retained as genes. However, those specific predictions of either program were retained only after performing a BLAST search (18) against the non-redundant (nr) database at NCBI (19) and finding hits in other species (Figure 2). As Figure 2 (a) illustrates, when we performed a BLAST search for the strain *Pyrococcus abyssi* GE5 (NC_000868), we excluded the genus *Pyrococcus* (taxid:2260) from the nr database. This logic simulates the factual case when annotating an anonymous genome because, in that case, the nr database will not contain any genes from itself and relative species.

Supplementary Table S3 lists predicted results for the joint application and for the two individual programs. Among the 50 genomes, 33 combined applications had a higher sensitivity than either ZCURVE or Glimmer independently, whereas 44 had lower additional positive rate than either program. When considering both indexes, 27 genomes had both an increased sensitivity and a decreased additional positive rate. Such results indicate that, in most cases, the joint prediction decreases the total gene number but contains more true genes. On average, the sensitivity increased 0.59% and 0.64% relative to ZCURVE and Glimmer, respectively. At the same time, the additional positive rate decreased 1.3% and 3.0% relative to ZCURVE and Glimmer, respectively. No predictions for any genome were worse when the two indexes were combined. In total, the joint application finds 740 and 724 more true genes than ZCURVE and Glimmer, respectively, in the 50 genomes. Furthermore, the false positive predictions decrease 2052 and 3954, respectively compared with the two single programs. Therefore, it is obvious that the joint application improves the prediction by finding more genuine genes and simultaneously decreasing the false-positive predictions when integrating results with the BLAST operation. However, the single method will not reach this purpose even though when integrating Blast operation. In fact, ZCURVE 3.0 could also be jointly used with any other gene-finding programs to improve the prediction result. On the basis of the above analysis, we strongly suggest that annotating researchers use multiple *ab initio* gene finders to minimize annotating errors. Many researchers have adopted such tactics. For example, in well-annotated genomes such as *Tistrella mobilis* KA081020-065 (20), *Amycolatopsis mediterranei* U32 (21), and five new strains of *Staphylococcus aureus* (22), the authors used both ZCURVE and Glimmer. The pipeline illustrated in Figure 2(b) may be used as one way to combine the two gene finders.

### Performance of predicting essential genes

In previous assessments of Geptop, annotated genes were used as the initial gene set (or input). Here, the set of predicted genes was taken as the initial set when it was integrated as the newly added function of ZCURVE 3.0. To evaluate the performance of the integrated program, seven bacteria were used in which genome-wide essentiality was determined after the publishing date of Geptop. As Table 2 (left columns) shows, the sensitivity ranged from 48.9% to 79.0%, whereas the specificity was 89.3% to 95.0% when Geptop was integrated into ZCURVE 3.0. The more reliable index, overall accuracy, which combines the effects from positive and negative samples, had an average value near 90%. Hence, our program could accurately predict essentiality in bacteria. We retained a very high specificity but significantly lower sensitivity because the number of non-essential genes was so large that even a quite low error rate of negative samples may cause many false-positive predictions; this result is not expected, particularly when the task is related to drug target discovery. Note that these seven genomes should be taken as independent tests because Geptop was developed two years ago and now it remains the original version. There is another possible cause of lower sensitivity in our prediction. Experimental results may also own bias to some extent because of deviation in optimal growing conditions or other factors. For example, for the three genomes with the lowest AUC in previous work, protein–protein association feature analysis suggested that our prediction may be not as bad as apparently illustrated (11). To our knowledge, there are no other programs that have the ability to automatically predict essential genes. In addition to dividing them into two types of genes, essential and non-essential genes, our program can also give the probability value for each gene being essential. This feature will be particularly useful for bacterial pathogens because new drug target genes against the pathogen may be identified from the list of predicted essential genes or conditional essential genes (23). A newly sequenced genome can be annotated using ZCURVE 3.0 to find both protein-coding genes and select essential genes.

**Table 2.** Prediction results of essential genes in seven genomes

Organism name	Essential gene number	Non-essential gene number	*S_n_*	*S_p_*	OA
*Bacteroides thetaiotaomicron* VPI-5482	325	4453	0.489	0.930	0.900
*Burkholderia thailandensis* E264	406	5226	0.623	0.907	0.887
*Salmonella typhimurium* SL1344	353	4093	0.790	0.929	0.918
*Shewanella oneidensis* MR-1	402	3663	0.689	0.960	0.933
*Sphingomonas wittichii* RW1	535	4344	0.385	0.946	0.884
*Burkholderia pseudomallei* K96243	505	5213	0.372	0.893	0.847
*Bacteroides fragilis* 638R	547	3703	0.395	0.950	0.879
Average			0.534	0.931	0.893

*S_n_*, sensitivity; *S_p_*, specificity; OA, overall accuracy.

## CONCLUSION

We updated ZCURVE for *ab initio* gene finding in prokaryotes. A higher accuracy was illustrated with the latest version, and it can give close accuracy with the commonly used program Glimmer 3.02. As the most prominent advantage, ZCURVE 3.0 can automatically select essential genes from the list of protein-coding genes, whereas none of the other *ab initio* gene-finding programs can provide such convenience. We hope that this feature will assist with identifying drug targets to select against pathogens. To facilitate its use, we provided a web-based automatic service for ZCURVE 3.0. The users can use the web service to analyze genomes without providing any user information.

## AVAILABILITY

http://cefg.uestc.edu.cn/zcurve/ or http://tubic.tju.edu.cn/zcurveb/.

## SUPPLEMENTARY DATA

Supplementary Data are available at NAR Online.

SUPPLEMENTARY DATA
