# Supplementary material for: ZCURVE 3.0: identify prokaryotic genes with higher accuracy as well as automatically and accurately select essential genes
Source: Nucleic Acids Res. 2015 May 14;43(Web Server issue):W85–90. doi: 10.1093/nar/gkv491 (PMC4489317; doi:10.1093/nar/gkv491)
Supplement: SUPPLEMENTARY DATA [file supp_43_W1_W85__index.html]

ZCURVE 3.0: identify prokaryotic genes with higher accuracy as well as automatically and accurately select essential genes — SUPPLEMENTARY DATA 

# ZCURVE 3.0: identify prokaryotic genes with higher accuracy as well as automatically and accurately select essential genes

## SUPPLEMENTARY DATA

- SUPPLEMENTARY DATA
- SUPPLEMENTARY DATA
- SUPPLEMENTARY DATA
- SUPPLEMENTARY DATA
