# Supplementary material for: ZCURVE 3.0: identify prokaryotic genes with higher accuracy as well as automatically and accurately select essential genes
Source: Nucleic Acids Res. 2015 May 14;43(Web Server issue):W85–90. doi: 10.1093/nar/gkv491 (PMC4489317; doi:10.1093/nar/gkv491)
Supplement: SUPPLEMENTARY DATA [file supp_gkv491_nar-00558-web-b-2015-File004.docx]

**Table S1**. Predictions for each of the 422 prokaryotic genomes by three programs.

**Table S2**. Predictions for each of the 2787 prokaryotic genomes by ZCURVE 3.0.

**Table S3.** Results of the joint application of ZCURVE 3.0 and Glimmer 3.02 with 50 prokaryotic genomes.
